# Diosmetin Alleviates MRSA-Induced Pneumonia in Mice by Inhibiting NLRP3 Inflammasome Activation and NF-κB Signaling Pathway

**DOI:** 10.3390/ph19050674

**Published:** 2026-04-25

**Authors:** Chenxi Wu, Huiguo Xie, Xiaofei Liang, Lujie Yang, Zhengxiao Ren, Ping Wu, Yingying Zhang

**Affiliations:** 1 School of Pharmacy, Shandong University of Traditional Chinese Medicine, Jinan 250355, China; wuchenxi1008@126.com (C.W.); 18870783345@163.com (H.X.); liangxiaofei200011@163.com (X.L.);; 2 Clinical Laboratory, Changqing District People’s Hospital, Jinan 250355, China

**Keywords:** diosmetin, methicillin-resistance *Staphylococcus aureus*, pneumonia

## Abstract

**Background/Objectives:** Methicillin-resistant *Staphylococcus aureus* (MRSA) is a multidrug-resistant pathogen that poses a major public health concern. It predominantly infects immunocompromised individuals and is frequently associated with severe pulmonary complications, including acute lung injury. Diosmetin, a natural flavonoid, known for its anti-inflammatory, antioxidant, and anti-infective properties. Nevertheless, its therapeutic mechanism in the treatment of acute pneumonia induced by MRSA remains unclear. **Methods:** In this study, we employed network pharmacology and molecular docking to elucidate the mechanisms underlying the therapeutic effect of diosmetin against MRSA-induced pneumonia. An MRSA pneumonia model was established in Balb/c mice. The impacts of diosmetin on murine pneumonia were evaluated by detecting biochemical indicators via HE staining, ELISA, RT-qPCR, and WB. In vitro experiments utilized RAW264.7 macrophages to establish an MRSA infection model for further validation of the therapeutic mechanisms of diosmetin. **Results:** In vivo results demonstrated that diosmetin alleviated MRSA-induced lung injury and reduced mortality by inhibiting the release of pro-inflammatory cytokines. Furthermore, compared with model mice, diosmetin-treated mice showed reduced phosphorylation levels of NLRP3, pro-caspase-1, ASC, and NF-κB p65, along with an increased level of IκBα in lung tissue. In vitro experiments indicated that diosmetin effectively reduced the levels of pro-inflammatory cytokines in MRSA-infected RAW264.7 macrophages and exerted anti-inflammatory effects by modulating the expression of NLRP3, pro-caspase-1, ASC, IκBα, and NF-κB p65. **Conclusions:** Our results demonstrate that diosmetin alleviates MRSA-induced pneumonia in mice, and this protective effect is achieved through dual inhibition of the NF-κB/NLRP3 inflammasome axis.

## 1. Introduction

*Staphylococcus aureus* is a Gram^+^ pathogenic [1] coccus that commonly colonizes humans. The wide clinical application of methicillin has led to natural selection of resistant stains and emergence of MRSA [2,3]. MRSA is one of the most significant pathogens causing both hospital-acquired and community-acquired pneumonia. Currently, the primary treatment strategy for MRSA pneumonia involves using antibiotics to eliminate the pathogen [4]. However, MRSA infection not only causes direct bacterial damage but also induces an upregulation of macrophage-derived inflammatory mediators due to high bacterial load, triggering excessive inflammatory responses. Simultaneously, the immune system recruits a large number of immune cells to the infection site, which may lead to secondary lung injury during bacterial clearance [5]. Relying solely on antibiotic therapy is far from sufficient. Therefore, combining anti-inflammatory treatment with antibacterial measures may hold greater clinical significance.

**Figure 1:**
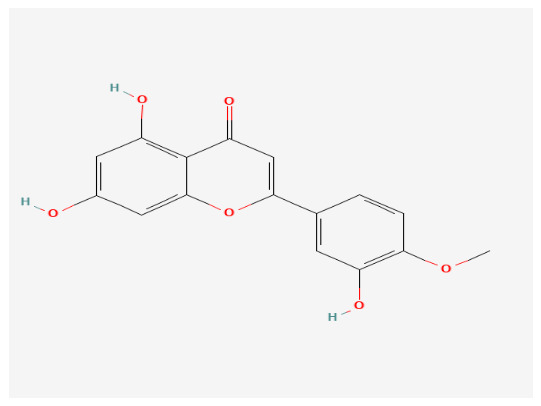
The chemical structural formula of diosmetin.

The anti-inflammatory activity of natural flavonoids is widely recognized. Diosmetin (5,7,3′-dihydroxy-4′-methoxy flavone, chemical structure shown in Figure 1), a flavonoid compound found in Nepeta and various traditional Chinese medicines.

Its mechanism of action is probably linked to the regulation of key inflammatory pathway. Experimental evidence indicates that diosmetin possesses anti-inflammatory properties across diverse models, largely by suppressing NF-κB signaling [6,7,8,9]. NF-κB pathway serves as a central regulatory hub in inflammatory responses. NF-κB is bound to IκB, and retained in the cytoplasm when nothing happened. In response to PAMPs, IκB undergoes phosphorylation and degradation. This allows NF-κB—primarily the p65/p50 heterodimer—to translocate rapidly into the nucleus, where it initiates the transcriptional expression of numerous downstream pro-inflammatory mediators [10].

The transcriptional activation of NF-κB exerts widespread effects. On one hand, it directly drives the synthesis of IL-6 and TNF-α [11,12]. On the other hand, NF-κB activation provides an essential signal for the assembly of NLRP3 inflammasome, namely by upregulating the expression levels of NLRP3 protein and pro-IL-1β [13,14]. NLRP3 inflammasome, a crucial multiprotein complex sensor within cells, can be activated by MRSA toxins or danger signals. Upon activation, it recruits and cleaves caspase-1, ultimately triggering the maturation and release of inflammatory factors. This process represents a core executive step in the amplification cascade of inflammatory responses [15].

We speculate that the inhibition of diosmetin on the NF-κB pathway carries dual anti-inflammatory significance: it directly reduces the production of pro-inflammatory cytokines and also indirectly restricts the full activation of NLRP3 inflammasome and the subsequent processing. This dual-level regulatory action on the NF-κB/NLRP3 inflammatory axis may represent an important molecular basis for diosmetin to alleviate excessive inflammatory responses and exert immunomodulatory functions, thereby providing a theoretical foundation for its potential intervention in MRSA pneumonia.

Based on the above background, this study aims to provide experimental evidence for the anti-inflammatory effects of diosmetin in MRSA pneumonia treatment. This study investigated the therapeutic mechanisms of diosmetin against Staphylococcus aureus pneumonia using network Pharmacology. The PPI network identified several core targets, including TNF-α, IL-6, NFKBIA, PTGS2, SRC, IL-1β, EGFR, MMP9, and CASP1. GO and KEGG enrichment analyses indicated that diosmetin may attenuate inflammatory responses through targeting of the NF-κB/NLRP3 axis. The docking result showed that diosmetin could bind strongly to IL-1β, CASP1, NFKBIA, IL-18, and NLRP3. Therefore, we propose the scientific hypothesis that diosmetin may effectively curb the excessive inflammatory response in MRSA pneumonia by regulating the NF-κB/NLRP3 inflammasome axis. To validate this hypothesis, we established a murine model of MRSA pneumonia and an MRSA-infected RAW264.7 cell model. The therapeutic effect of diosmetin was systematically evaluated both in vitro and in vivo, and its underlying mechanism was thoroughly investigated.

## 2. Results

### 2.1. Screening of Potential Targets for Diosmetin Treating MRSA Pneumonia

**Figure 2:**
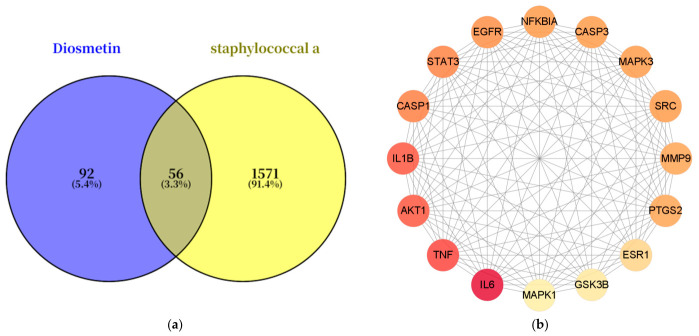
Analysis of the core targets of diosmetin against SA pneumonia. (**a**) A Venn diagram shows 56 intersection targets between diosmetin and SA pneumonia. (**b**) The core therapeutic targets (defined as the intersection of the top 16 genes ranked by degree, closeness, and betweenness centrality via the CentiScape plugin) were identified from PPI network. The depth of the target color is positively correlated with its degree value.

Network pharmacology was employed to systematically predict the therapeutic targets and mechanisms of diosmetin against *Staphylococcus aureus* pneumonia. Potential targets of diosmetin were retrieved from the TCMSP database, yielding 151 candidates. Concurrently, 1627 targets associated with *S. aureus* pneumonia were collected from the GeneCards database. A comparative analysis identified 56 overlapping targets, which were considered as the putative targets for diosmetin’s action against this infection Figure 2a.

These 56 overlapping targets were analyzed using Cytoscape. Topological analysis revealed that several proteins, including TNF, IL-6, NFKBIA, PTGS2, SRC, IL-1β, EGFR, MMP9, and CASP1, functioned as central hub nodes within the PPI network Figure 2b, suggesting their critical roles in the potential therapeutic effects.

**Figure 3:**
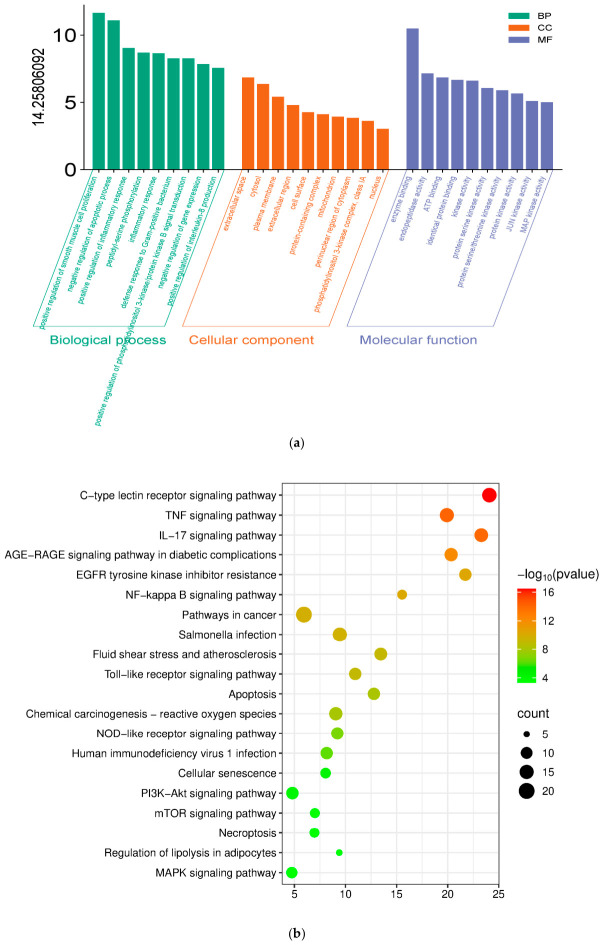
Functional and pathway analysis of diosmetin in the treatment of SA pneumonia via GO and KEGG enrichment. (**a**) GO enrichment results across BP, CC, MF categories. (**b**) Key signaling pathways identified by KEGG enrichment analysis. The horizontal axis represents the gene ratio, while the vertical axis represents the enriched pathway name. The color scale indicates different thresholds of the *p*-value, and the size of the dot indicates the number of genes corresponding to each pathway.

To clarify the underlying BP, CC, MF and signaling pathways, GO and KEGG enrichment analyses were performed on the 56 common targets. The GO analysis demonstrated pronounced enrichment in biological processes pertaining to inflammatory response, cytokine production, and response to bacterium Figure 3a. Complementing this, KEGG pathway analysis revealed a marked enrichment in key inflammatory signaling cascades, most notably the NF-κB/NLRP3 axis Figure 3b. These data collectively suggest that the anti-pneumonia effect of diosmetin may be mediated through the modulation of these critical inflammatory pathways.

### 2.2. Molecular Docking of Diosmetin with Key Proteins

**Figure 4:**
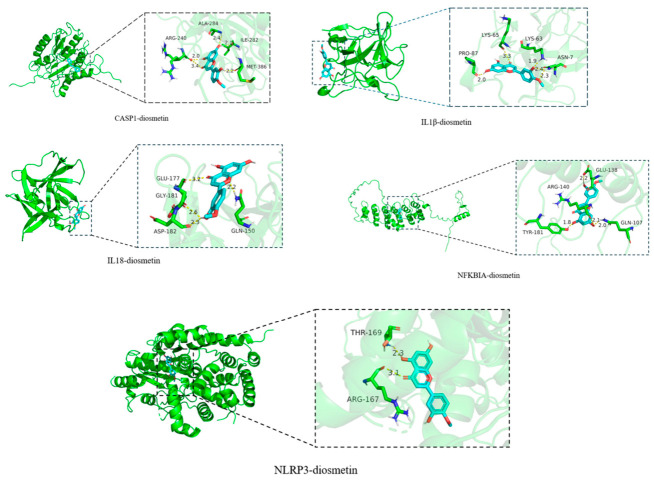
Docking results of diosmetin with the targets.

Drawing on the findings from network pharmacology analysis and a comprehensive review of the literature, five key proteins (NFKBIA, NLRP3, CASP1, IL-18, IL-1β) involved in the *Staphylococcus aureus* pneumonia pathway were selected for Molecular docking with diosmetin. Results are shown in Figure 4 and Table 1. All five complexes demonstrated favorable binding affinity, with binding energies of less than −5.0 kcal/mol, indicating strong ligand–receptor, where a lower binding energy correlates with a higher binding affinity.

### 2.3. Diosmetin Alleviates the MRSA-Induced Lung Injury

**Figure 5:**
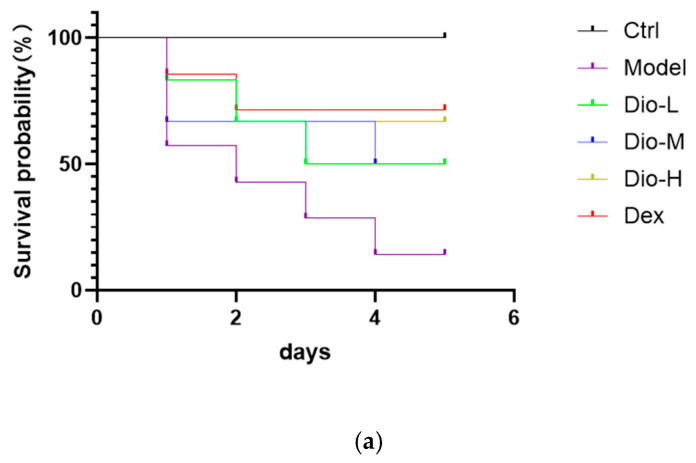

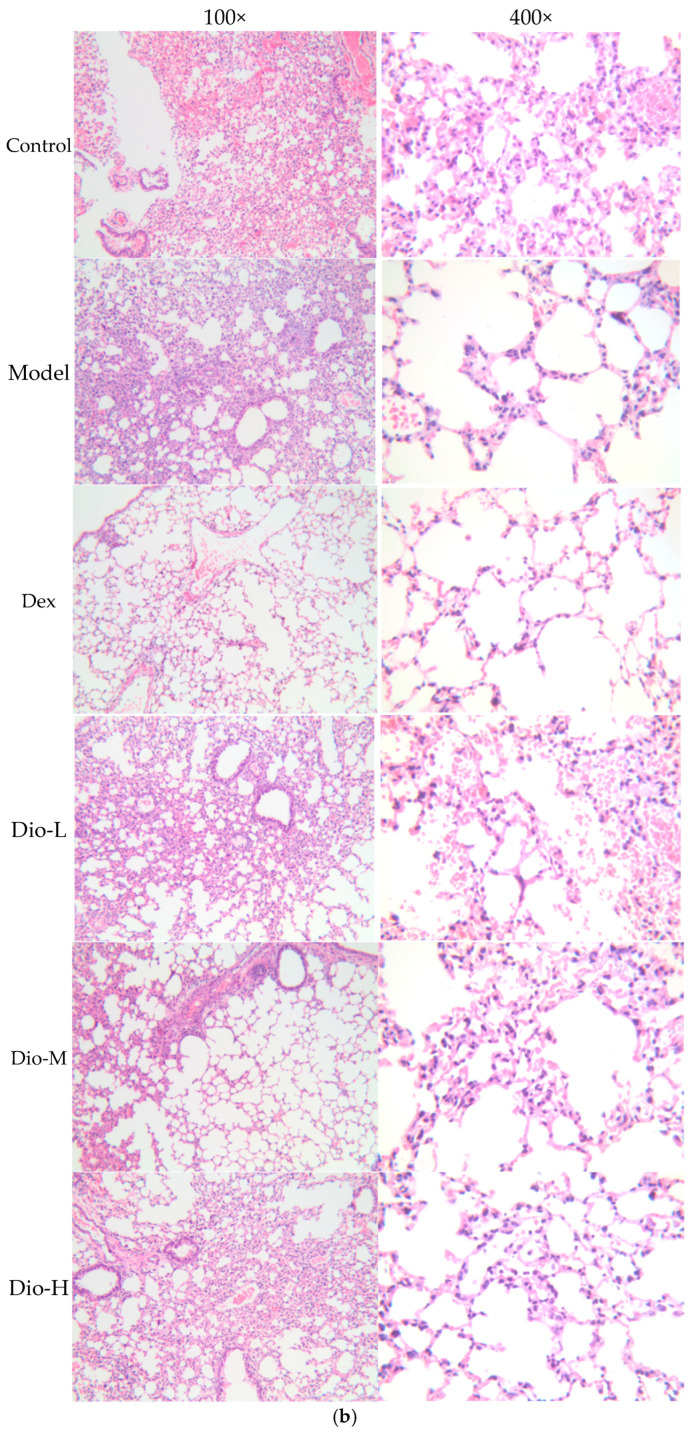
(**a**) Mice were treated with medication three days after MRSA infection and monitored daily. (**b**) The H&E histopathological analysis of MRSA-infected mice (original magnification 100× and 400×).

After treatment with diosmetin, the survival rate of the mice increased compared to the model group, indicating that diosmetin can improve the survival rate of mice after MRSA infection. Pathological examination revealed that lung tissue sections from the model group mice exhibited typical pathological alterations of acute pneumonia: severe disruption of alveolar architecture, markedly widened septa, extensive inflammatory cell infiltration and red proteinaceous exudate within alveolar spaces, along with focal hemorrhages in some areas. In contrast, lung sections from mice treated with diosmetin showed significant mitigation of pathological injury. The alveolar structure remained relatively intact, with reduced thickening and disorganization of alveolar septa. The number of inflammatory cells infiltrating the alveolar spaces was notably decreased, and both exudation and hemorrhage were markedly attenuated, see Figure 5.

### 2.4. Diosmetin Diminished Pro-Inflammatory Cytokine Production Following MRSA

#### Lung Infection

**Figure 6:**
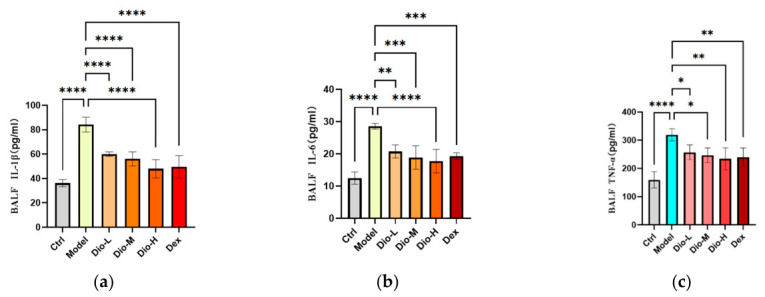
Diosmetin attenuates inflammatory factors production in mice after MRSA infection. The levels of inflammatory factors in the BALF of mice in the Ctrl, model, Dio-L (25 mg/kg), Dio-M (50 mg/kg), Dio-H (100 mg/kg) and Dex (5 mg/kg) were measured by ELISA. (**a**) IL-1β; (**b**) IL-6; (**c**) TNF-α; *n* = 6 ± SEM, * *p* < 0.05, ** *p* < 0.01, *** *p* < 0.001, **** *p* < 0.0001.

To investigate the regulation of MRSA infection on cytokine secretion in vivo, the concentrations of IL-6, TNF-α, and IL-1β in BALF supernatant were measured at 24 h post-infection using a sandwich ELISA. Compared with the control group, the expression of IL-6, TNF-α, and IL-1β were markedly elevated in the model group, indicating a heightened inflammatory state. In contrast, diosmetin (25, 50, 100 mg/kg) dose-dependently inhibited the release of these inflammatory factors compared to the model group, suggesting a potent anti-inflammatory effect Figure 6. These data demonstrate that diosmetin can effectively suppress the release of inflammation-related cytokines in MRSA-infected mice.

### 2.5. Diosmetin Downregulated the mRNA Expression of Related Genes In Vivo Following MRSA Infection

**Figure 7:**
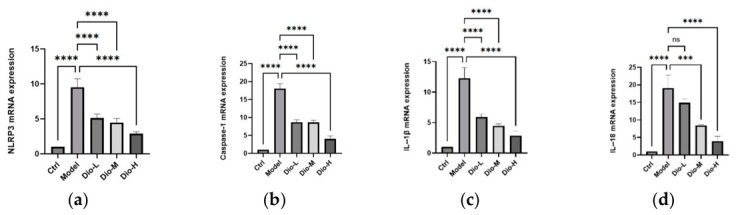
The mRNA levels of NLRP3 inflammasome pathway-related genes in lung tissue of Ctrl, model, Dio-L (25 mg/kg), Dio-M (50 mg/kg) and Dio-H (100 mg/kg) mice. (**a**) NLRP3, (**b**) Caspase-1, (**c**) IL-1β, and (**d**) IL-18. (*n* = 3; *** *p* < 0.001, **** *p* < 0.0001 ns indicates no significant difference).

The experimental results are presented in Figure 7. The mRNA expression of NLRP3, Caspase-1, IL-18, as well as IL-1β, was markedly upregulated in the lung tissue of the model group compared to the control group, indicating the activation of inflammatory pathways. By contrast, the diosmetin treatment markedly reduced the mRNA expression of these gene compared with the model group. These results demonstrate that diosmetin can inhibit genes transcription involved in the NLRP3 inflammasome in mice lung tissue, exhibiting a clear anti-inflammatory effect.

### 2.6. Diosmetin Suppresses the Expression of NF-κB p65, IκBα, NLRP3, ASC, and Pro-Caspase-1 In Vivo Following MRSA Lung Infection

**Figure 8:**
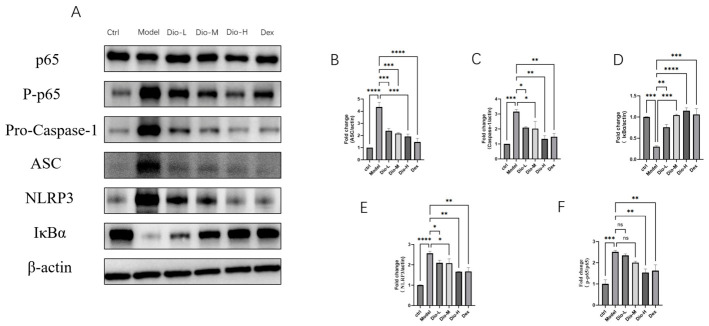
Effects of DIO on the expression of key proteins in the NF-κB/NLRP3 axis in lung tissue of mice with inflammatory model. (**A**) Representative Western blot results showing protein bands. (**B**–**F**) Bar charts showing the relative quantitative analysis of protein expression: (**B**) ASC, (**C**) Pro-Caspase-1, (**D**) IκBα, and (**E**) NLRP3 (all normalized to β-actin), and (**F**) the ratio of phospho-p65 to p65. The experiment included six groups: Ctrl, Model, Dio-L (25 mg/kg), Dio-M (50 mg/kg), Dio-H (100 mg/kg), and Dex (5 mg/kg). Data were presented as fold change (*n* = 3 ± SEM; * *p* < 0.05, ** *p* < 0.01, *** *p* < 0.001, **** *p* < 0.0001; ns indicates no significant difference).

The expression levels of key proteins in the NF-κB/NLRP3 pathway were assessed by Western blotting Figure 8. While the total protein level of p65 exhibited no significant change, the phosphorylation level of p65 (p-p65) was markedly upregulated in the model group relative to the control group. Concurrently, the expression levels of NLRP3, pro-caspase-1, and ASC were significantly increased, whereas the total protein level of IκBα was decreased in the model group. These findings suggest that IκBα underwent phosphorylation and degradation, leading to the activation of the NF-κB pathway, which in turn promotes NLRP3 inflammasome assembly and ultimately triggers an inflammatory response. Diosmetin treatment led to a progressive reduction in the levels of p-p65, NLRP3, pro-caspase-1, and ASC, along with a concomitant increase in IκBα levels, compared with the model group. This indicates that diosmetin can inhibit the phosphorylation of IκBα and p65, as well as the expression of NLRP3, pro-caspase-1, and ASC, resulting in the suppression of the NF-κB/NLRP3 axis.

### 2.7. Diosmetin Reduces the Levels of Inflammatory Factors in Macrophages Infected with MRSA In Vitro

Macrophages perform various functions such as the modulation of immunity. As a result, they are frequently employed as an important model for evaluating the immunomodulatory capacity.

**Figure 9:**
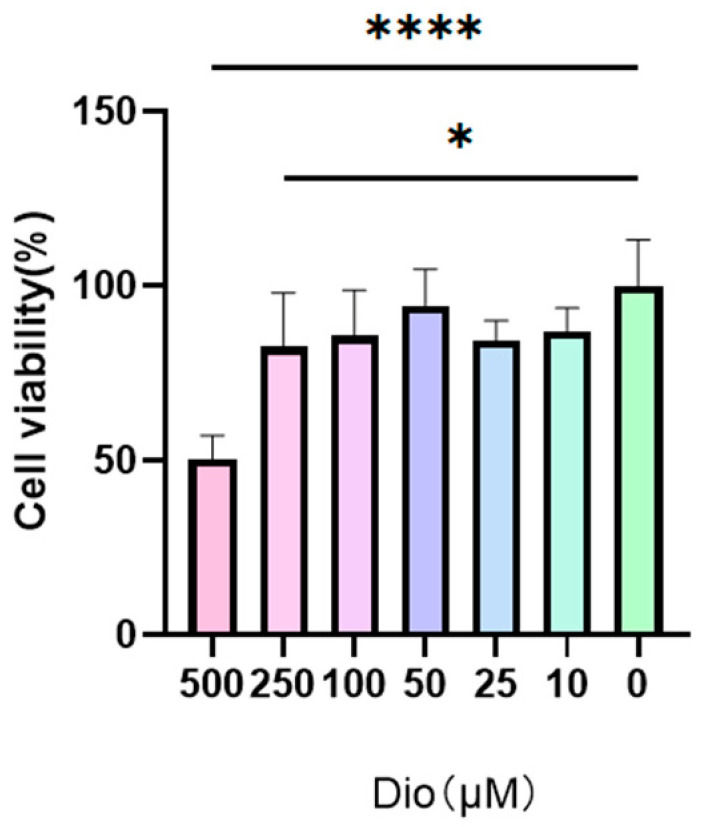
Results of the cytotoxicity of diosmetin against RAW264.7 cells. (*n* = 3 ± SEM; * *p* < 0.05, **** *p* < 0.0001).

The cytotoxicity of diosmetin toward RAW264.7 cells is presented in Figure 9. The results indicate that diosmetin exhibits no cytotoxicity at concentrations up to 250 μM.

**Figure 10:**
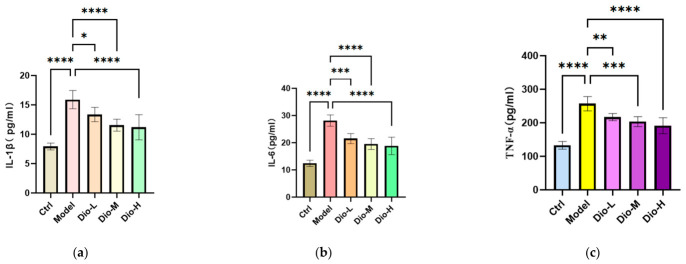
Effects of DIO on pro-inflammatory cytokine levels in the RAW264.7 cell model infected with MRSA. Ctrl, model, Dio-L (25 μM), Dio-M (50 μM) and Dio-H (100 μM) (**a**) IL-1β; (**b**) IL-6; (**c**) TNF-α; *n* = 6 ± SEM; * *p* < 0.05, ** *p* < 0.01, *** *p* < 0.001, **** *p* < 0.0001.

Subsequently, the effect of diosmetin on cytokine production was evaluated in an MRSA-infected macrophage model. The expression of IL 6, TNF α, and IL 1β were markedly increased in the culture supernatant of the model group relative to the control group. Conversely, the diosmetin treatment significantly decreased the release of these inflammatory cytokines, see Figure 10. These findings indicate that diosmetin modulated pro-inflammatory cytokine production in RAW264.7 cells following MRSA infection and can effectively inhibit the secretion of inflammation-related cytokines.

### 2.8. Diosmetin Inhibits the Expressions of NF-κB p65, IκBα, NLRP3, ASC, and Pro-Caspase-1 in MRSA-Infected Macrophages In Vitro

**Figure 11:**
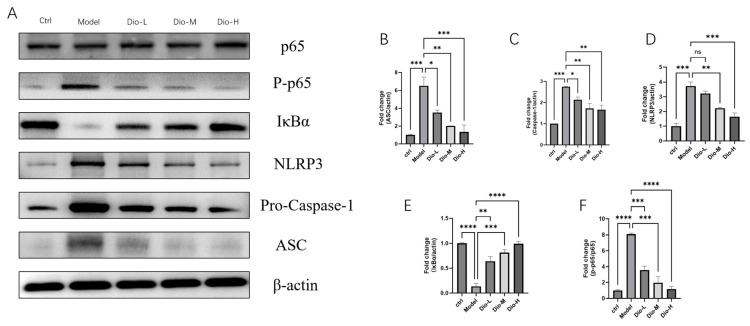
The expression of proteins in the RAW264.7 cell model infected with MRSA treated with DIO. (**A**) Representative Western blot results showing protein bands (**B**–**F**). Bar charts showing the relative quantitative analysis of protein expression levels: (**B**) ASC, (**C**) Pro-Caspase-1, (**D**) NLRP3, and (**E**) IκBα (all normalized to β-actin), and (**F**) the ratio of phospho-p65 to p65. The experiment included six groups: Ctrl, Model, Dio-L 25 μM, Dio-M 50 μM, Dio-H 100 μM. (*n* = 6 ± SEM; * *p* < 0.05, ** *p* < 0.01, *** *p* < 0.001, **** *p* < 0.0001; ns indicates no significant difference).

To further investigate the effects of diosmetin on the NF-κB/NLRP3 inflammasome axis, the expression of NF-κB p65, IκBα, NLRP3, ASC and Pro-Caspase-1 in MRSA-infected macrophages treated with diosmetin were measured by Western blot. Consistent with in vivo findings, diosmetin reduced the expression of NLRP3, ASC, and Pro-Caspase-1 and the phosphorylation of NF-κB p65 in RAW264.7 cells. This indicates that diosmetin interferes with MRSA-infected macrophages by suppressing the NF-κB/NLRP3 axis. Figure 11.

## 3. Discussion

MRSA pneumonia is one of the common types of hospital-acquired and community-acquired pneumonia, accounting for 13% of nosocomial pneumonia cases in Asia, with a mortality rate that can even reach 40.8% [16]. The core pathology of MRSA pneumonia involves bacterial infection-induced lung tissue damage and excessive inflammation, including infiltration of immune cells such as neutrophils and macrophages [17], as well as the overproduction of inflammatory cytokines like interleukins and tumor necrosis factor [18,19] and the activation of key inflammatory signaling pathways such as NF-κB and NLRP3 [20]. While antibiotics directly target the pathogen, natural products that modulate the host’s immune response offer a complementary therapeutic strategy by mitigating excessive inflammation [21]. Previous studies have demonstrated that Isoacteoside, Verbascoside and Echinacoside extracted from BCLF Decoction exerts therapeutic effects against MRSA pneumonia through a dual mechanism: by inhibiting the activity of the *S. aureus* virulence factor SrtA and the expression of Hla as well as by modulating the TNF-α/TNFR1/NF-κB/MMP9 axis [22]. Echinacoside, a polyphenolic compound derived from natural products, in combination with vancomycin, inhibits the activity of the SrtA, thereby offering a therapeutic approach for MRSA pneumonia [23]. Decursinol Angelate, extracted from *Angelica gigas*, ameliorates MRSA infection by modulating the pro-inflammatory functions of macrophages [5]. Ginsenoside Rb1, extracted from *Panax ginseng*, protects against *S*. *aureus* pneumonia in mice by inhibiting the activation of the TLR-2-mediated NF-κB and MAPK pathways [24]. Previous studies have identified the therapeutic of flavonoids for the treatment of pneumonia [25]. This study investigated the molecular mechanisms by which the flavonoid diosmetin modulates MRSA pneumonia. Through network pharmacology, we predicted the targets of diosmetin in the treatment of *Staphylococcus aureus* pneumonia. Molecular docking results further demonstrated that diosmetin can stably bind to several key proteins, including NFKBIA, NLRP3, CASP1, IL-18, and IL-1β. Therefore, to validate this hypothesis, the present study performed verification in a murine model of MRSA pneumonia as well as in an in vitro macrophage infection model.

Previous studies have demonstrated that MRSA infection can bind to TLRs and recruit MyD88, leading to the activation of the NF-κB pathway [18,26,27]. After entering the nucleus, NF-κB interacts with the NLRP3, resulting in a significant upregulation of NLRP3 protein expression. Subsequently, triggered by other intracellular signals, the NLRP3 protein undergoes conformational changes and assembles with ASC and caspase-1 to form the inflammasome, which combats infection and clears damage [15]. Additionally, MRSA infection can also be driven by its virulence factor HlgB, which transmits signals through the host proteins AMFR and TAB3, ultimately activating NF-κB pathway via TAK1 to generate pro-inflammatory cytokines and exacerbate inflammation [28]. Many drugs have been proven to inhibit inflammatory responses both in vivo and in vitro by suppressing NF-κB/NLRP3 axis [29,30,31,32,33]. Diosmetin is one of them. Our study found that it can inhibit the activation of NF-κB by stabilizing IκBα and suppressing p65 phosphorylation, and it can also regulate downstream factors (IL-1β,IL-6 and TNF-α). This suggests that diosmetin reduces the phosphorylation of IκBα by inhibiting upstream kinase activity. Due to decreased phosphorylation, the degradation of IκBα is also reduced, leading to the accumulation of stable, non-phosphorylated IκBα total protein in the cytoplasm. As a result, NF-κB is more “locked” in the cytoplasm, unable to translocate into the nucleus, thereby inhibiting the subsequent release of inflammatory factors. Meanwhile, diosmetin significantly downregulates the protein expression of NLRP3, ASC, and Pro-Caspase-1, as well as reduces the production of the downstream product mature IL-1β, thereby alleviating excessive inflammatory responses.

Preliminary experiments and references indicated that diosmetin does not exhibit direct bactericidal activity at normal concentrations [34]. However, the literature suggests that diosmetin can reduce the pathogenicity of SA by inhibiting the expression of Hla in SA [35], and as the concentration of diosmetin increases, LDH levels gradually decrease [36]. This indicates that eugenol does not function directly as an antibacterial agent; its value lies not in replacing antibiotics but in mitigating the lung damage caused by MRSA infection through its anti-inflammatory mechanism, thereby alleviating pneumonia symptoms induced by MRSA and buying time for the body to recover while the bacteria persist.

However, constrained by time and a series of other factors, we only investigated the anti-inflammatory effects of diosmetin alone, without combining it with antibiotics effective against MRSA such as vancomycin, which deviates from clinical practice. Moreover, research on the molecular targets of diosmetin remains preliminary, and further exploration of its molecular mechanisms should be conducted in future studies.

In summary, our study found that diosmetin exerts its anti-inflammatory effects in the treatment of MRSA pneumonia by modulating the NF-κB pathway, thereby regulating downstream NLRP3 inflammasome and other inflammatory cytokines.

## 4. Materials and Methods

### 4.1. Network Pharmacology

To systematically explore the potential therapeutic targets of diosmetin against *Staphylococcus aureus* pneumonia, network pharmacology was employed. Potential targets of diosmetin were retrieved from the TCMSP database [37]. Concurrently, disease-associated targets for *S. aureus* pneumonia were collected from the GeneCards database using “staphylococcus aureus pneumonia” as the keyword [38,39]. The intersection of diosmetin and *staphylococcus aureus* pneumonia targets was identified and visualized by the Venny 2.1.0 online tool. Common targets were then imported into Cytoscape (3.10.4) software to construct a “diosmetin-target-MRSA pneumonia” network. To further elucidate the PPI landscape, the intersection targets were submitted to STRING website, and the resulting PPI network was analyzed within Cytoscape to identify core targets based on topological features [40]. Finally, GO (covering BP, CC and MF) and KEGG analysis were executed on the intersection targets using DAVID database to predict the underlying biological mechanisms and signaling pathways.

### 4.2. Molecular Docking

The binding affinity of diosmetin to key proteins identified from the network analysis was evaluated via molecular docking. The three-dimensional structure of diosmetin was obtained from PubChem. The crystal structures of target proteins (NFKBIA, CASP1, NLRP3, IL-1β, IL-18) were acquired from the PDB and prepared using PyMOL 2.5 software. And simulations were carried out using AutoDock Tools 1.5.6. The target proteins were saved in PDBQT. Docking simulations were performed with AutoDock Vina to calculate binding energies, PyMOL 2.5 visualized the result [41].

### 4.3. Reagents and Antibodies

Diosmetin (Batch No. 520-34-3) was provided by Anhui Kuer Bioengineering Co., Ltd. Dexamethasone (Cat# HY-14648) was sourced from MedChemExpress (Shanghai, China). ELISA kits for quantifying were obtained from Jiangsu Enzyme Immunoindustrial Co., Ltd. (Yancheng, China). Primary antibodies against GAPDH (GB15004), NF-κB p65 (GB11997), phospho-p65 (GB113882), NLRP3 (GB114320), β-actin (GB15003) and IκBα (GB151509) were purchased from Servicebio (Wuhan, China). Caspase-1 antibody was acquired from ABclonal (Wuhan, China), and the ASC antibody (Bs-41334R) was from Bioss (Beijing, China). Goat anti-rabbit IgG (A0208) was obtained from Beyotime (Shanghai, China).

### 4.4. RAW264.7 Cell Culture and Viability Assay

The RAW264.7 cell (Procell, Wuhan, China) was cultured and preserved in DMEM containing 10% FBS, 1% P/S. To assess cytotoxicity, RAW264.7 cells were seeded in 96-well plates at a density of 1 × 10^5^ cells/well and treated with varying concentrations of diosmetin (0, 10, 25, 50, 100, 250, 500 μM) for 12 h, then determined using the MTT assay, measuring OD at 450 nm.

### 4.5. MRSA Infection

MRSA (ATCC 43300) from frozen stocks (−80 °C) was revived on solid agar plates, followed by culture in liquid medium. The concentration was adjusted to 10^7^–10^8^ CFU/mL prior to infection. RAW264.7 cells, after adaptation in antibiotic-free DMEM, were infected with MRSA (MOI = 10) for 3 h. Following infection, cells were treated with diosmetin (low 25 μM, medium 50 μM, and high dose 100 μM) for more 3 h. Subsequently, cell culture supernatants were harvested for ELISA, and cells were harvested for protein expression analysis.

### 4.6. Animals and Drug Treatment

This animal protocol was approved by the Animal Ethics Committee of Shandong University of Traditional Chinese Medicine (No. SDUTCM20250711002) and in accordance with China’s laboratory animal management laws. Balb/c mice (18 ± 2 g) were housed under SPF conditions. A mouse model of MRSA pneumonia was established by daily intranasal instillation of 20 μL of a bacterial suspension (10^8^ CFU/mL) for three consecutive days under isoflurane anesthesia. We randomized the mice to the following groups (*n* = 6 per group): normal control, model (MRSA-infected), positive control (dexamethasone 5 mg/kg), and diosmetin-treated (low 25 mg/kg, medium 50 mg/kg, and high dose 100 mg/kg). Drug administration began three days post-infection. Mouse health status and survival were monitored every 12 h for 96 h.

### 4.7. Histological Assessment

Following collection, lung tissues were fixed in 4% paraformaldehyde, processed into paraffin-embedded blocks, and sectioned. The resulting sections were stained with H&E to visualize general cellular architecture and pathological changes. Pathological changes were evaluated and scored by a blinded observer under a light microscope at 100× and 400× magnification. Six random fields per section from six mice per group were analyzed.

### 4.8. Elisa Assay

The concentrations of inflammatory cytokines in mouse BALF and RAW264.7 cell culture supernatants were assessed using meimian ELISA kits refer to the manual for specific experimental procedures. The OD was measured at 450 nm using a microplate reader.

### 4.9. PCR Assay

Total RNA was extracted from lung tissues using an RNA extraction kit and reverse-transcribed into cDNA [42]. Then proceed with RT-qPCR. The mRNA expression of genes were calculated using the 2^−ΔΔCt^ method. All specific primers used are listed in a Table 2.

### 4.10. Western Blotting Assay

Lung tissue and cellular pellets were lysed in RIPA lysis buffer. Equivalent quantities of protein were resolved on 10% SDS-PAGE and electrophoretically transferred onto PVDF membranes [43], then blocked with 5% non-fat milk and probed with primary antibodies for 13 h at 4 °C. Following washes, the membranes were incubated secondary antibodies. The blots were detected using an ECL reagent [44] and captured with a Tanon-4800 MULTI imaging system. Quantitative analysis of band density was performed with ImageJ (1.54) software.

### 4.11. Statistical Analysis

Continuous data are presented as the mean ± SEM. Statistical evaluations were performed using GraphPad Prism software (Version 10.0). Where appropriate, inter-group comparisons were conducted by one-way ANOVA followed by Dunnett’s post hoc test for multiple comparisons. A *p*-value of less than 0.05 was defined as statistically significant.

## Figures and Tables

**Table 1:** The binding energy of diosmetin to the target.

	CASP1	IL1B	IL18	NFKBIA	NLRP3
	8WRA	5R8Q	4HJJ	AF_P25963_F1	8WSM
Diosmetin	−8.6	−7.8	−6.2	−5.8	−9.1

**Table 2:** Specific primers used for PCR amplification.

Primer Name	Sequence (5′-3′)
IL-1β-F	TCGCAGCAGCACATCAACAAG
IL-1β-R	TCCACGGGAAAGACACAGGTAG
CASP1-F	CGAGGGTTGGAGCTCAAGTT
CASP1-R	AGAAGTCTTGTGCTCTGGGC
NLRP3-F	AGGAGGAAGAAGAAGAGAGGAGAGG
NLRP3-R	TTGAGAAGAGACCACGGCAGAAG
IL-18-F	ACTTTGGCCGACTTCACTGT
IL-18-R	CCTCGAACACAGGCTGTCTT
GAPDH-F	TGATGGGTGTGAACCACGAG
GAPDH-R	AGTGATGGCATGGACTGTGG

## Data Availability

The original contributions presented in this study are included in the article. Further inquiries can be directed to the corresponding authors.

## Abbreviations

The following abbreviations are used in this manuscript:

MRSA	Methicillin-resistant *Staphylococcus aureus*
Dio	Diosmetin
TCMSP	Traditional Chinese Medicine Systems Pharmacology Database
PPI	protein–protein interaction
BP	Biological Process
CC	Cellular component
MF	Molecular Function
GO	Gene Ontology
KEGG	Kyoto Encyclopedia of Genes and Genomes
BALF	bronchoalveolar lavage fluid
Dex	Dexamethasone
H&E	Hematoxylin and Eosin
NF-κB	Nuclear Factor kappa-B
IL-1β	Interleukin-1β
IL-6	Interleukin-6
IL-18	Interleukin-18
TNF-α	tumor necrosis factor-α
NLRP3	NOD-like receptor thermal protein domain associated protein 3
ASC	apoptosis-associated speck-like protein containing a caspase recruitment domain
SrtA	Sortase A
Hla	Alpha-hemolysin
TLRs	Toll-like receptors
MyD88	Myeloid differentiation primary response protein 88
HlgB	Gamma-hemolysin component B
AMFR	Autocrine motility factor receptor
TAB3	TAK1-binding protein 3
TAK1	TGF-β-activated kinase 1
